# Serum IgG4 level during initial treatment as a predictor of relapse in IgG4-related disease

**DOI:** 10.1371/journal.pone.0282852

**Published:** 2023-03-09

**Authors:** Su Jin Choi, Soo Min Ahn, Ji Seon Oh, Seokchan Hong, Chang-Keun Lee, Bin Yoo, Yong-Gil Kim

**Affiliations:** 1 Department of Rheumatology, Ulsan University Hospital, University of Ulsan College of Medicine, Ulsan, South Korea; 2 Department of Rheumatology, Asan Medical Center, University of Ulsan College of Medicine, Seoul, South Korea; 3 Department of Information Medicine, Asan Medical Center, Seoul, South Korea; Ajou University School of Medicine, REPUBLIC OF KOREA

## Abstract

**Introduction:**

We aimed to investigate the predictors of relapse in immunoglobulin G4-related disease (IgG4-RD), focusing on the serum IgG4 levels during initial treatment.

**Methods:**

We retrospectively recruited 57 patients with IgG4-RD who were treated with immunosuppressants and elevated serum IgG4 levels in a tertiary hospital between January 2011 and December 2020. They were followed up for ≥ 6 months after initiation of immunosuppressive therapy. Clinical and laboratory findings including serum IgG4 levels (reference value: 6–121 mg/dL) were compared between relapsed (*n* = 13) and non-relapsed (*n* = 44) groups. Multivariate Cox regression analysis was used to assess the predictors for relapse. We performed a Kaplan–Meier analysis with a log-rank test to evaluate the cumulative relapse rate for two years.

**Results:**

Median serum IgG4 levels at baseline were 321 mg/dL in the relapsed group and 299 mg/dL in the non-relapsed group. Serum IgG4 levels were normalized after six months in five (38.5%) relapsed and 28 (63.6%) non-relapsed patients. In multivariate Cox regression analysis, the normalization of serum IgG4 levels at six months was associated with a lower risk of relapse, with a hazard ratio of 0.232 (*p* = 0.019). Central nervous system involvement was associated with the relapse, with a hazard ratio of 21.130 (*p* = 0.015). The cumulative relapse rate for two years was lower in the normal serum IgG4 group at six months than in the elevated serum IgG4 group at six months (*p* = 0.027).

**Conclusion:**

Our study suggests that normalization of serum IgG4 levels during immunosuppressive treatment for IgG4-RD independently predicts relapse-free outcomes. Thus, monitoring serum IgG4 levels might be used as a marker of prognosis.

## Introduction

Immunoglobulin G4-related disease (IgG4-RD) is a systemic fibro-inflammatory condition of unknown etiology [1]. This disorder is characterized by lymphoplasmacytic tissue infiltration with IgG4-positive plasma cells and elevation of serum IgG4 levels. Diffuse swelling, mass formation, or fibrosis can occur in most organs, and the pancreas, biliary tree, lacrimal gland, salivary gland, and retroperitoneum/aorta are frequently involved [2]. The mainstay treatment for the achievement of remission is a glucocorticoid, and most patients respond well to the initial treatment. However, patients often experience relapses during glucocorticoid tapering or maintenance therapy [3]. In patients with a high risk of relapse, more stringent treatment and follow-up strategies may be considered to prevent frequent relapse, organ damage, and additional glucocorticoid use. However, prognostic markers are still challenging in IgG4-RD.

Polyclonal hypergammaglobulinemia is related to various diseases including liver disease, autoimmune disease, infection, and malignancy [4]. Patients with IgG4-RD exhibit polyclonal hypergammaglobulinemia with beta-gamma bridging, which is sometimes mistaken for the increased polyclonal IgA levels in liver disease. In IgG4-RD, elevated polyclonal serum IgG4 levels are associated with an abundant lymphoplasmacytic infiltration of IgG4-positive plasma cells. Serum IgG4 levels are known to increase in about 43%–90% of patients with IgG4-RD [5, 6] and are validated clinical tools for diagnosis and activity assessment [7, 8]. As for the determination of relapse risk, several studies have suggested baseline serum IgG4 levels as the risk factor [9, 10]. Nonetheless, there is a lack of consensus on whether serial changes in serum IgG4 levels are associated with disease relapse. Most IgG4-RD patients who initiate the treatment exhibit decreased serum IgG4 levels, with B-cell depletion [11, 12]. However, serum IgG4 levels are frequently not normalized despite improving clinical course during glucocorticoid tapering. We can speculate that incomplete reduction in serum IgG4 levels during treatment reflects the remaining activity of IgG4-RD, which may contribute to the disease relapse later [13]. However, little is known about the association between the disease relapse and the change of serum IgG4 levels in patients with IgG4-RD. Therefore, in this study, we investigated the clinical parameters associated with the relapse in IgG4-RD, focusing on the changes of serum IgG4 levels during treatment.

## Materials and methods

### Patients

This study was conducted on patients with active IgG4-RD who were treated with immunosuppressants and had elevated serum IgG4 levels at a tertiary referral center in South Korea between January 2011 and December 2020. All patients met the 2011 IgG4-RD diagnostic criteria [7]: (1) a clinical examination with diffuse/localized swelling or masses in single or multiple organs; (2) an elevated serum IgG4 level; and (3) histopathologic findings of lymphoplasmacytic infiltration and fibrosis, > 40% IgG4-positive plasma cells, and > 10 IgG4-positive plasma cells per high-power field. Patients were also evaluated according to the 2019 American College of Rheumatology (ACR)/European League Against Rheumatism (EULAR) classification criteria for diagnosis of IgG4-RD [14]. Patients with pancreatic involvement were classified as definite or probable cases using the International Consensus Diagnostic Criteria (ICDC) for autoimmune pancreatitis [15]. We excluded patients with other rheumatic diseases, malignancy, or infection. Either glucocorticoid alone or glucocorticoid plus azathioprine were used as an initial regimen for remission and were maintained for at least six months and up to 24 months. To evaluate the factors associated with relapse, we classified patients into relapsed and non-relapsed groups.

### Data collection

We retrospectively reviewed the electronic medical records of these patients at the time of the initial treatment, including age, sex, diagnostic classification, disease duration, affected organs, and treatment regimen. The IgG4-RD responder index (RI), which is a score for each organ system and serum IgG4 level, was calculated to assess the disease activity [8]. Laboratory parameters such as erythrocyte sedimentation rate (reference value: 0–20 mm/hr) and the levels of C-reactive protein (reference value: 0–0.6 mg/dL), serum IgG (reference value: 700–1,600 mg/dL), and serum IgG4 (reference value: 6–121 mg/dL) were also collected. Serum IgG levels were measured using nephelometry (Nephelometer, Dade Behring, Germany) and serum IgG4 levels were measured using a single radial immunodiffusion method (The Binding Site, Birmingham, UK).

At six months of initial treatment, the clinical and serological response was assessed. The clinical response included the IgG4-RD RI score, > 50% decline in IgG4-RD RI score, and remission. Remission was defined as meeting all of the following [16]: (1) > 50% decline in IgG4-RD RI score; (2) tapering of prednisolone to less than 10 mg/day; and (3) no relapse during the initial treatment period (within six months). Relapse was defined as recurrence, worsening, or de novo organ involvement as determined via imaging or the analysis of biochemical parameters (e.g., urinalysis and liver function), regardless of serum IgG4 levels. The change of serum IgG4 levels at six months was also evaluated as the serological response. Patients were divided into two groups according to serum IgG4 levels at six months (normalized and elevated serum IgG4 levels).

### Statistical analysis

Continuous variables were represented as median (interquartile range [IQR]) and analyzed using the Mann–Whitney *U* test. Categorical data were expressed as numbers (percentages) and compared using the chi-squared test and Fisher’s exact test. Wilcoxon signed-rank test was used to compare paired data of serum IgG4 levels within the two groups at baseline and six months. We evaluated the sensitivity, specificity, positive predictive value (PPV), and negative predictive value (NPV) of normalized serum IgG4 level for the prediction of relapse. Univariate and multivariate Cox proportional hazard models were conducted to assess the hazard ratio (HR) and 95% confidence interval (CI) for the predictor of relapse in IgG4-RD. Multivariate Cox proportional hazard models include variables with a p-value < 0.2 in the univariate Cox analysis, and were adjusted for age, sex, baseline IgG4-RD RI score, baseline serum IgG4 level, and medication. We performed the Kaplan–Meier analysis with the log-rank test to evaluate the cumulative relapse rates for two years in groups with normalized and elevated serum IgG4 levels at six months. A *p*-value of < 0.05 was considered to be statistically significant. We used SPSS software, version 24.0, for all analyses.

### Ethical consideration

This study was conducted in accordance with the Declaration of Helsinki principles and was approved by the Institutional Review Board of Asan Medical Center (Seoul, South Korea) (IRB number: 2020–1771). The Institutional Review Board of Asan Medical Center waived informed consent because of the retrospective nature of the study.

## Results

### Baseline characteristics of entire patients

We enrolled a total of 57 active IgG4-RD patients with elevated serum IgG4 levels at baseline. According to the 2011 diagnostic criteria of IgG4-RD, 24 (42.1%) and 33 (57.9%) patients were diagnosed with definite and possible cases, respectively. Forty (70.2%) patients fulfilled the 2019 ACR/EULAR classification criteria. Forty-five (78.9%) patients were male, and the median age at the start of treatment was 61 (IQR, 55–67) years. The median IgG4-RD RI score was 12 (IQR, 10–19), and the organ distribution was as follows: pancreas (66.7%), lymph node (52.6%), retroperitoneum/aorta (29.8%), biliary tree (28.1%), salivary gland (26.3%), lacrimal gland (17.5%), kidney (15.8%), lung (10.5%), and central nervous system including meninges (3.5%). According to the ICDC for autoimmune pancreatitis, 32 (84.2%) of 38 patients with pancreatic involvement were definitive cases, and six (15.8%) were probable cases. The initial dose of prednisolone was median 30 (IQR, 30–40) mg/day. Median serum IgG4 levels at baseline were 305 (IQR, 192–555) mg/dL. Thirty-seven (64.9%) patients showed serum IgG4 levels ≥ 2x the upper normal of limit (UNL), of which 12 (21.1%) patients had serum IgG4 levels ≥ 5x the UNL. The median follow-up duration was 17.2 (IQR, 12.3–24.0) months. Thirteen (22.8%) patients experienced relapse, with a median time to relapse of 16.8 (IQR, 9.8–20.0) months.

### Baseline characteristics and treatment response in relapsed and non-relapsed groups

The baseline characteristics of the relapsed and non-relapsed groups are summarized in Table 1. Most clinical features, including male, age, involved organs, IgG4-RD RI score, and initial median prednisolone dose, did not differ between the two groups. There was no significant difference in the median levels of serum IgG4 between relapsed and non-relapsed groups (321 [IQR, 254–580] mg/dL vs. 299 [IQR, 173–546] mg/dL, *p* = 0.309). The proportion of patients with serum IgG4 levels greater than twice the UNL was also not different between relapsed and non-relapsed groups (76.9% vs. 61.4%, *p* = 0.346).

**Table 1:** Baseline characteristics of relapsed and non-relapsed cases in Korean patients with IgG4-related disease.

Variable	Relapse	No relapse	*p*-value
	(*n* = 13)	(*n* = 44)	
**Clinical characteristics**			
Male	12 (92.3%)	33 (75.0%)	0.261
Age (years)	59 (52–70)	61 (55–66)	0.768
Time since diagnosis (months)	0.2 (0.1–15.2)	0.2 (0.1–3.5)	0.931
IgG4-RD criteria			
Definite	6 (46.2%)	18 (40.9%)	0.736
Possible	7 (53.8%)	26 (59.1%)	0.736
Organ involvement			
Pancreas	9 (69.2%)	29 (65.9%)	1.000
Bile duct	5 (38.5%)	11 (25.0%)	0.483
Lymph node	5 (38.5%)	25 (56.8%)	0.244
Retroperitoneum/aorta	3 (23.1%)	14 (31.8%)	0.734
Lacrimal gland	3 (23.1%)	7 (15.9%)	0.680
Salivary gland	2 (15.4%)	13 (29.5%)	0.478
Kidney	2 (15.4%)	7 (15.9%)	1.000
Lung	1 (7.7%)	5 (11.4%)	1.000
Central nervous system	1 (7.7%)	1 (2.3%)	0.407
Multi-organ involvement (≥ 3)	6 (46.2%)	20 (45.5%)	0.965
IgG4-RD RI score	15 (10–21)	12 (10–18)	0.787
Initial prednisolone dose (mg/day)	30 (30–40)	30 (30–40)	0.630
**Laboratory findings**			
ESR (mm/hr)^a^	72 (9–82)	16 (10–49)	0.344
CRP (mg/dL)^b^	0.29 (0.10–1.11)	0.12 (0.10–0.43)	0.191
Serum IgG level (mg/dL)^c^	1,460 (1,295–2,225)	1,635 (1,370–1,850)	0.797
Serum IgG4 level (mg/dL)^c^	321 (254–580)	299 (173–546)	0.309
Range of serum IgG4 level			
> normal but < 2x UNL	3 (23.1%)	17 (38.6%)	0.346
≥ 2x UNL	10 (76.9%)	27 (61.4%)	0.346

IgG4-RD, IgG4-related disease; RI, responder index; ESR, erythrocyte sedimentation rate; CRP, C-reactive protein; UNL, upper normal of limit.

Data are expressed as median (interquartile range) or number (%).

^a^ ESR data (reference value: 0–20 mm/hr) were available for five patients of the relapsed group and 22 patients of the non-relapsed group.

^b^ Data on CRP levels (reference value: 0–0.6 mg/dL) were available for 12 patients of the relapsed group and 42 patients of the non-relapsed group.

^c^ Reference values of serum IgG and IgG4 levels were 700–1,600 mg/dL and 6–121 mg/dL, respectively.

Table 2 shows the treatment response at six months in the relapsed and non-relapsed groups. Both groups exhibited similar improvements in clinical items such as median IgG4-RD RI score (relapsed, 4 [IQR, 2–7] vs. non-relapsed, 3 [IQR, 1–5], *p* = 0.324) and acquisition of remission (69.2% vs. 56.8%, *p* = 0.423). The treatment regimen, including prednisolone and/or azathioprine, did not differ between the two groups. In terms of serological changes, the median serum IgG4 levels declined at six months in both groups (relapsed, from 321 [IQR, 254–580] to 169 [IQR, 98–222] mg/dL, *p* = 0.001; non-relapsed, from 299 [IQR, 173–546] to 86 [IQR, 49–193] mg/dL, *p* < 0.001). However, the relapsed group showed higher median serum IgG4 levels at six months than the non-relapsed group (*p* = 0.033). Likewise, the proportion of patients with normalized serum IgG4 levels at six months tended to be low in the relapsed group compared with that of the non-relapsed group (38.5% vs. 63.6%, *p* = 0.106). When evaluating the performance of normalized serum IgG4 levels for the relapse-free outcome, the sensitivity and specificity were 63.6% and 61.5%, respectively. While the PPV was 84.8%, the NPV was 33.3%.

**Table 2:** Clinical and serological response at six months of treatment in relapsed and non-relapsed patients.

Variable	Relapse	No relapse	*p*-value
	(*n* = 13)	(*n* = 44)	
IgG4-RD RI score	4 (2–7)	3 (1–5)	0.324
50% reduction in IgG4-RD RI score	11 (84.6%)	38 (86.4%)	1.000
Remission	9 (69.2%)	25 (56.8%)	0.423
Serum IgG4 level (mg/dL) ^a^	169 (98–222)	86 (49–193)	0.033
Normalized serum IgG4 level	5 (38.5%)	28 (63.6%)	0.106
Prednisolone dose (mg/day)	10.0 (5.0–12.5)	10.0 (5.0–15.0)	0.898
Azathioprine	3 (23.1%)	10 (22.7%)	1.000

IgG4-RD RI, IgG4-related disease responder index.

^a^ Reference value of serum IgG4 levels was 6–121 mg/dL.

Data are expressed as median (interquartile range) or number (%).

### Predictors of disease relapse

We performed Cox regression analyses to identify the risk factors for relapse in our cohort of IgG4-RD patients (Table 3). In the univariate Cox regression analysis, normalized serum IgG4 level at six months (HR = 0.297; 95% CI, 0.096–0.923; *p* = 0.036) was associated with the relapse-free outcome. In addition, central nervous system involvement (HR = 8.737; 95% CI, 0.908–84.031; *p* = 0.061) was possible factor associated with the relapse. After adjusting for age, sex, baseline IgG4-RD RI score, baseline serum IgG4 level, prednisolone dose, and azathioprine use, the multivariate analysis revealed that central nervous system involvement (HR = 21.130; 95% CI, 1.828–244.208; *p* = 0.015) was associated with an increased risk of relapse. In contrast, normalized serum IgG4 level at six months was significantly associated with a lower risk of relapse (HR = 0.232; 95% CI, 0.069–0.784; *p* = 0.019).

**Table 3:** Univariate and multivariate Cox regression analyses for predictors of relapse.

Variable	Univariate analysis		Multivariate analysis	
	HR (95% CI)	*p*-value	HR (95% CI)	*p*-value
**Baseline clinical characteristics**				
Male	3.225 (0.419–24.835)	0.261		
Age (years)	0.987 (0.924–1.054)	0.696		
Time since diagnosis (months)	1.015 (0.989–1.041)	0.257		
Organ involvement				
Pancreas	0.792 (0.241–2.598)	0.700		
Bile duct	1.846 (0.603–5.648)	0.283		
Lymph node	0.578 (0.189–1.769)	0.337		
Retroperitoneum/aorta	0.707 (0.194–2.573)	0.599		
Salivary gland	0.651 (0.144–2.951)	0.578		
Lacrimal gland	2.090 (0.568–7.694)	0.268		
Kidney	1.497 (0.316–6.925)	0.619		
Lung	0.629 (0.082–4.843)	0.657		
Central nervous system	8.737 (0.908–84.031)	0.061	21.130 (1.828–244.208)	0.015
Multi-organ involvement (≥ 3)	1.309 (0.438–3.912)	0.629		
IgG4-RD RI score	1.039 (0.932–1.158)	0.490		
Initial prednisolone dose (mg/day)	1.001 (0.991–1.011)	0.882		
Serum IgG4 level (mg/dL)	1.001 (1.000–1.002)	0.250		
**At 6 months**				
IgG4-RD RI score	1.045 (0.913–1.195)	0.524		
Remission	1.871 (0.575–6.086)	0.298		
Normalized serum IgG4 level	0.297 (0.096–0.923)	0.036	0.232 (0.069–0.784)	0.019
Prednisolone dose (mg/day)	1.012 (0.937–1.092)	0.768		
Azathioprine	0.688 (0.188–2.514)	0.572		

HR, hazard ratio; CI, confidence interval; IgG4-RD RI, IgG4-related disease responder index.

### Cumulative relapse rate in groups with normalized and elevated serum IgG4 levels at six months

**Fig 1:**
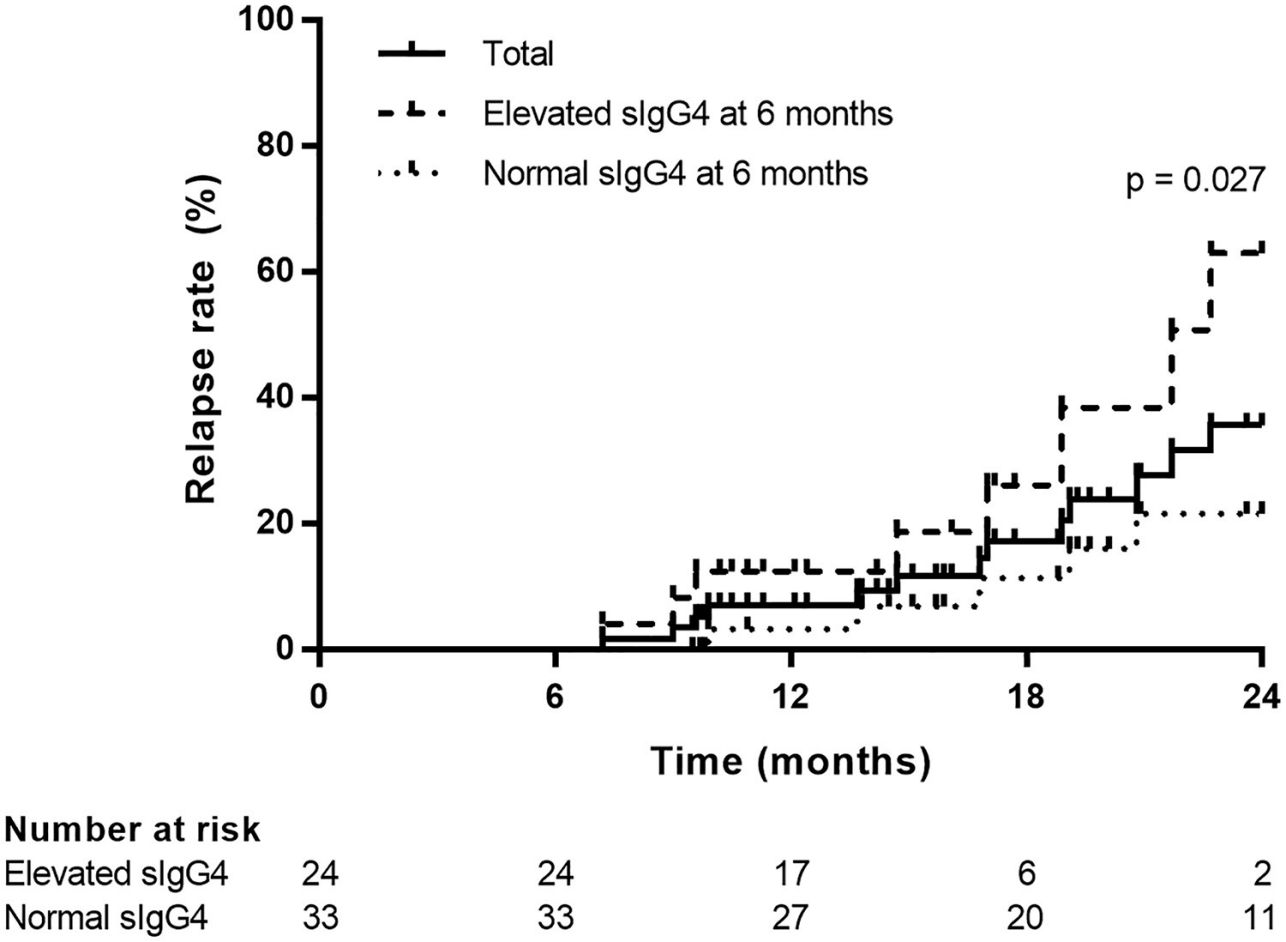
Kaplan-Meier curve for cumulative relapse rates in groups with normalized and elevated serum immunoglobulin G4 (IgG4) level at six months. Log-rank test was used to compare the cumulative relapse rates over two years in the groups with normalized and elevated serum IgG4 levels at six months. sIgG4, serum IgG4.

As shown in Fig 1, the overall cumulative relapse rates were 7.2% at 12 months and 35.7% at 24 months. When assessed according to the groups with normalized and elevated serum IgG4 levels at six months, the cumulative relapse rate at 12 and 24 months was 3.3% and 21.6% in the normalized group, and 12.5% and 63.1% in the elevated group, respectively. The elevated group showed a higher relapse rate than the normalized group (log-rank test, *p* = 0.027).

## Discussion

Identifying relapse risk in IgG4-RD is essential for maintaining a favorable outcome during the follow-up period. Although several prognostic factors have been suggested, the relationship between the relapse and the changes in serum IgG4 levels is not clear. We demonstrated that patients with normalized serum IgG4 levels at six months of treatment had a low risk of relapse in IgG4-RD. In contrast, central nervous system involvement was the risk factor associated with disease relapse. This study proposes the persistent elevation of serum IgG4 levels despite initial treatment as a predictor of the relapse in IgG4-RD.

We reported that approximately one-fourth of patients experienced relapse more than once over 24 months, and the overall cumulative relapse rate was 7.2% in 12 months and 35.7% in 24 months. Previous studies indicated the relapse rates of IgG4-RD ranging from 23% to 58%, depending on the disease characteristics and treatment regimen of the cohorts [9, 17]. In our study, only patients with elevated serum IgG4 levels were included, and about 20% of patients showed baseline serum IgG4 level ≥ 5x the UNL. The pancreas, biliary tree, lymph node, retroperitoneum/aorta, salivary gland, and lacrimal gland were the commonly affected organs. Some patients received a combination therapy of azathioprine and glucocorticoid. In IgG4-RD, various immunosuppressive agents including azathioprine, mycophenolate mofetil, and cyclophosphamide may be used as glucocorticoid-sparing agents. B-cell depletion therapy with rituximab is also an effective treatment option. In our study, azathioprine was the only immunosuppressive agent that had been administered. After six months of initial treatment, most patients had declined IgG4-RD RI scores and had reduced prednisolone to low doses. Considering the heterogeneous nature of IgG4-RD, our relapse rates seem reasonable compared to the previous studies.

There have been clinical prediction markers for the relapse of IgG4-RD, such as age, male, IgG4-RD RI score, blood eosinophils, and glucocorticoid regimen [18–20]. Elevation of baseline serum IgG4 level is also considered the risk factor for relapse [21]. However, the clinical implications of changes in serum IgG4 levels have been controversial. In a study by Liu *et al*., the increase in serum IgG4 levels by more than 20%, 50%, or 70% compared to remission was not associated with the relapse [22]. Further, the prospective UK cohort did not demonstrate an association between the relapse and the normalized serum IgG4 levels during or after treatment [9]. On the other hand, the study on autoimmune pancreatitis revealed a higher relapse rate in patients with persistently elevated serum IgG4 levels than in those with normalized levels after treatment [23]. Similar results were also found in other reports of IgG4-RD [24, 25]. This discrepancy raises the question about the clinical setting in which the change of serum IgG4 level helps predict the relapse. We found that normalized serum IgG4 level at six months of treatment predicted the relapse-free outcome. In previous studies of serial change in serum IgG4 levels, treatment generally led to an initial decrease in serum IgG4 levels even in the relapsed group [10, 11, 26]. However, during the 3–6 months of tapering glucocorticoid, serum IgG4 levels in the relapsed group tended to remain above the normal range or re-increase, whereas the non-relapsed group did not [10]. This difference may suggest that persistent elevation of serum IgG4 levels after initial treatment reflects remaining disease activity.

Despite the usefulness of serum IgG4 levels for diagnosis and disease activity in IgG4-RD, it is not fully understood what elevated serum IgG4 levels imply in the pathogenesis. However, in a study that used an adoptive transfer mouse model, the administration of IgG4 from patients with IgG4-RD resulted in the disease [27]. In a recent study, autoantibodies of the IgG4 and IgG1 subclasses against annexin A11 were detected in patients with IgG4-RD [28]. Additionally, IgG4 and IgE autoantibodies against galectin-3 correlate with increased IgG4 levels in IgG4-RD [29]. Since galectin-3 is associated with fibrosis, these antibodies may contribute to tissue fibrosis in IgG4-RD pathogenesis. Considering the pathogenic role of IgG4 in IgG4-RD, serum IgG4 levels are expected to be an indicator of IgG4-RD pathogenesis. Patients with high serum IgG4 levels tended to have more IgG4-related features in autoimmune pancreatitis pathology than those with low serum IgG4 levels [30]. Furthermore, serum IgG4 level is positively correlated with the number of circulating plasmablasts and T-follicular helper cells that play a crucial role in pathogenesis [31, 32]. In a study of circulating plasmablast in IgG4-RD patients treated with B-cell depletion therapy, the number of plasmablasts decreased after treatment, and then re-elevated in relapsed patients [26]. T-follicular helper cells also mediate the interaction between B cells and T cells and the induction of IgG4 [33]. Therefore, serum IgG4 level can be considered one of the variables representing T cell–B cell collaboration as an easily measurable indicator in the clinical setting because the decrease in serum IgG4 levels may be attributed to the loss of plasmablast and T-follicular helper cells. Further studies are needed to determine how to interpret changes in serum IgG4 levels from the pathogenesis of IgG4-RD.

This study also suggested central nervous system involvement such as meningitis as a risk factor of the relapse. Clinical and laboratory features and prognosis of IgG4-RD may vary depending on the involved organ [34–36]. Lanzillotta *et al*. reported that patients with head and neck involvement showed a favorable relapse-free outcome at 12 months compared to patients with other organ involvement [37]. Although pachymeningitis is a rare neurologic manifestation of IgG4-RD, with a prevalence of 2.4–4.1% [34, 38], a case-oriented review of IgG4-related pachymeningitis showed a relapse rate of 42.1% after discontinuation of glucocorticoid treatment [39]. Considering that the patients included in this review had relatively low serum IgG4 levels and often showed isolated manifestation without systemic involvement, the relapse rate of patients with pachymeningitis tended to be relatively high. Our study also showed an association between central nervous system involvement and relapse of IgG4-RD, indicating that close monitoring of disease activity is critical in IgG4-RD patients with central nervous system involvement.

There are some limitations to this study. First, this was a retrospective study conducted in a single center. There may have been informational bias in the medical records that could affect the results. For example, some laboratory parameters were not measured, including gamma-globulins via serum protein electrophoresis. Next, we included only the patients with elevated serum IgG4 levels at baseline to evaluate the significance of normalized serum IgG4 levels. Although most patients in previous studies also showed elevated serum IgG4 levels, baseline serum IgG4 level itself is associated with the disease relapse. However, given that patients with normal serum IgG4 levels were excluded, the prognostic value of change in serum IgG4 level may have been further strengthened. This point should be considered when interpreting the results of this study. Finally, we defined relapse as recurrence or worsening as determined via imaging findings or the deterioration of biochemical parameters. Clinical symptoms were not used in the definition of relapse, though additional evaluations including via imaging were performed if relapse was suspected. Nevertheless, a possible underestimation of relapsed cases cannot be excluded.

In conclusion, serum IgG4 level is a useful marker to diagnose and predict the IgG4-RD. Although there is interest in the clinical implication of a condition with persistently elevated serum IgG4 levels, the association with relapse is not clear. This study showed that serum IgG4 level during initial treatment was significantly associated with relapse of IgG4-RD. Patients whose serum IgG4 level has not been normalized after initial treatment should be monitored for the disease activity during the follow-up period.

## Supporting information

S1 Data (XLSX)
